# The role of atrial natriuretic peptide to attenuate inflammation in a mouse skin wound and individually perfused rat mesenteric microvessels

**DOI:** 10.14814/phy2.12968

**Published:** 2016-09-26

**Authors:** Fitz‐Roy E. Curry, Joyce F. Clark, Yanyan Jiang, Min‐Ho Kim, Roger H. Adamson, Scott I. Simon

**Affiliations:** ^1^ Department of Physiology & Membrane BiologyUniversity of California, DavisDavisCalifornia; ^2^ Department of Biomedical EngineeringUniversity of California, DavisDavisCalifornia; ^3^ Present address: Department of Biological SciencesKent State UniversityKentOhio

**Keywords:** Individually perfused microvessels, leukocyte deformability, leukocyte–endothelial interaction, vascular permeability

## Abstract

We tested the hypothesis that the anti‐inflammatory actions of atrial natriuretic peptide (ANP) result from the modulation of leukocyte adhesion to inflamed endothelium and not solely ANP ligation of endothelial receptors to stabilize endothelial barrier function. We measured vascular permeability to albumin and accumulation of fluorescent neutrophils in a full‐thickness skin wound on the flank of LysM‐EGFP mice 24 h after formation. Vascular permeability in individually perfused rat mesenteric microvessels was also measured after leukocytes were washed out of the vessel lumen. Thrombin increased albumin permeability and increased the accumulation of neutrophils. The thrombin‐induced inflammatory responses were attenuated by pretreating the wound with ANP (30 min). During pretreatment ANP did not lower permeability, but transiently increased baseline albumin permeability concomitant with the reduction in neutrophil accumulation. ANP did not attenuate acute increases in permeability to histamine and bradykinin in individually perfused rat microvessels. The hypothesis that anti‐inflammatory actions of ANP depend solely on endothelial responses that stabilize the endothelial barrier is not supported by our results in either individually perfused microvessels in the absence of circulating leukocytes or the more chronic skin wound model. Our results conform to the alternate hypothesis that ANP modulates the interaction of leukocytes with the inflamed microvascular wall of the 24 h wound. Taken together with our previous observations that ANP reduces deformability of neutrophils and their strength of attachment, rolling, and transvascular migration, these observations provide the basis for additional investigations of ANP as an anti‐inflammatory agent to modulate leukocyte–endothelial cell interactions.

## Introduction

The primary homeostatic action of atrial natriuretic peptide (ANP) in the microcirculation is to increase endothelial barrier permeability to plasma proteins when plasma volume is increased. These microvascular actions act in parallel with the well‐known renal functions as a natriuretic and diuretic. Recent published data has drawn attention to additional actions of ANP which may be vasoprotective. These include actions of ANP as a vasodilator to improve perfusion and as a modulator of the immune system. The combination of functions as a vasodilator, diuretic, and possible immune modulator has increased interest in the use of ANP analogs to improve vascular functions in a clinical setting. For example, in Japan there are ongoing clinical trials in patients with various forms of acute and chronic heart failure (Suwa et al. 2005; Mitaka et al. 2011; Mori et al. 2014). In addition, several investigations, particularly those using cultured endothelial cell monolayers as model systems, suggest ANP may activate anti‐inflammatory mechanisms: one line of investigation suggests that ANP may attenuate inflammatory responses via a variety of mechanisms, including stabilizing the endothelial cell cytoskeleton via cAMP, cGMP, and Rac/Rho activation under a range of experimental conditions (Klinger et al. 2006; Surapisitchat et al. 2007; Birukova et al. 2008; Xing and Birukova 2010); attenuating calcium influx (Kiemer et al. 2002; Furst et al. 2008; Chen et al. 2013); or reducing generation of proinflammatory agents (e.g., ROS) from activated endothelium, neutrophils, or mast cells or other inflammatory cells (Vollmar 2005; Kuhn 2012; Chen et al. 2013).

Another line of investigation focuses on the role of ANP in reducing leukocyte/endothelial interactions. Several studies suggest ANP limits activation of Nf‐kappa B, and reduces ischemic reperfusion injury (el Mtairag et al. 2002; Vollmar 2005; Ladetzki‐Baehs et al. 2007; Chujo et al. 2008). In our laboratory, we have recently demonstrated that ANP reduced accumulation of polymorphonuclear leukocytes (PMNs) in a mouse skin wound after exogenous thrombin was added to the wound (Morikis et al. 2015). In the latter study we demonstrated that the mechanism of action of ANP, added at concentrations within the physiological and pathophysiological range (1–10 nmol/L), involved an attenuation of PMN rolling, adhesion, and transendothelial migration by reducing their deformability and limiting the area of adhesive contact with the endothelium (Morikis et al. 2015). Despite the range of investigations on the relative influence of ANP on PMN‐dependent and endothelial‐dependent inflammation, studies of its affect on intact microvascular beds have been less well studied. There is a particular need for investigations of the action of ANP to modify vascular permeability and endothelial/leukocyte interactions evaluated simultaneously.

The aim of this study was to employ two established models of vascular permeability modulation to investigate the role of ANP in attenuating increased vascular permeability in microvessels under conditions where PMN interaction with the microvascular wall is detected and manipulated. One model is the full‐thickness skin wound on the back of lysM‐EGFP knock‐in mouse whose mature circulating PMN express EGFP. This model enables the measurement of both PMN accumulation and changes in permeability by whole animal fluorescence detection of labeled albumin simultaneously with the EGFP signal from the wound bed. We investigated the action of ANP to modulate thrombin‐induced hyperpermeability and PMN accumulation in a full‐thickness skin wound on the mouse flank. These experiments were performed under identical conditions as our published studies, where ANP was found to reduce PMN accumulation during acute thrombin activation of a 24‐h skin wound (Kim et al. 2009; Morikis et al. 2015). In a second in vivo model, we evaluated the action of ANP to modulate acute inflammatory responses to histamine and bradykinin addition in individually perfused rat venular microvessels with no prior exposure to inflammation. In these vessels blood was washed out of the vessel lumen at the beginning of the experiment so that the action of ANP was tested in the absence of arrested or rolling leukocytes.

Our results conform to the hypothesis that ANP may act as an anti‐inflammatory agent under conditions of sustained inflammation such as those in a full‐thickness skin wound. We conclude that the mechanism of action of ANP is primarily through modulation of leukocyte/endothelial interactions. This finding is consistent with in vitro experiments that reveal ANP acts to decrease PMN deformability and shear resistant arrest on inflamed endothelium in shear flow. Furthermore, ANP did not significantly attenuate an acute increase in permeability in individually perfused microvessels exposed to either bradykinin or histamine in the absence of leukocytes.

## Methods

### Animals

Mice stably expressing the lysM‐EGFP gene were generated by cross‐breeding 129Sv lysM‐EGFP mice (generously provided by Dr. Thomas Graf, Albert Einstein College of Medicine, Bronx, NY) with C57BL/6J (Jackson, Bar Harbor, ME) for at least nine generations in the animal facility at University of California at Davis and were housed in the same facility. Experiments in the 24 h wound were carried out in male and female mice between 8 and 12 weeks of age in all the experiments. Mice were anesthetized with an intraperitoneal injection of ketamine (80 mg/kg)‐xylazine (10 mg/kg), and the back skin hair was then removed with a mechanical shaver. After sterilization with 10% wt/vol povidine‐iodine and 70% alcohol, 6 mm in diameter circular full‐thickness wound was made using a skin biopsy punch (Robbins Instruments, Chatham, NJ). All mouse experiments were approved by the Institutional Animal Care and Use Committee of the University of California at Davis (animal protocol nos 11777 and 13386). Experiments to measure albumin permeability coefficients and hydraulic conductivity were carried out on male rats (Harlan Sprague–Dawley, 350–450 g); anesthetized with pentobarbital (100 mg/kg body wt. sc); and maintained with additional pentobarbital (30 mg/kg sc) as determined using toe‐pinch reflex. At the end of experiments animals were euthanized with saturated KCl. Animal protocols (no. 16158) were approved by the Institutional Animal Care and Use Committee of the University of California, Davis.

### In vivo whole animal fluorescence imaging of neutrophils and AlexaFluor‐BSA

**Figure 1:**
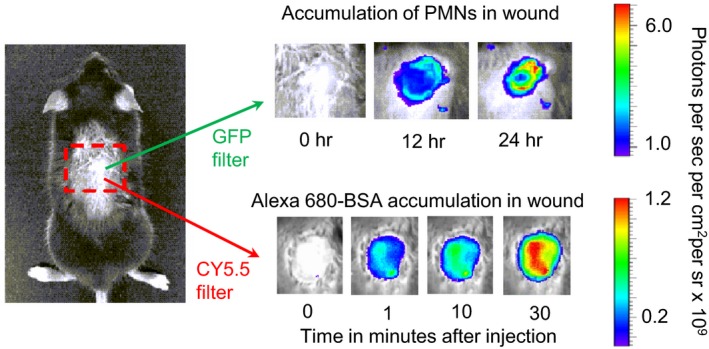
Schematic showing the methods to measure changes in either the fluorescence intensity (FI, photons/sec/cm^2^/sr) from either EGFP‐labeled polymorphonuclear leukocytes (PMNs) or albumin labeled with the long wavelength fluorescent Alexa‐680 in the back skin wound of an anesthetized mouse. The apparent permeability coefficient of the labeled albumin is measured from the step increase in FI as the tracer is injected into the tail vein and the subsequent increase in FI as the tracer accumulates in the tissue. This arrangement enabled us to demonstrate that atrial natriuretic peptide increases the apparent albumin permeability in the 24‐h skin wound under the same conditions that decreased PMNs in the wound and thrombin induced further accumulation in the 24 h wound (Morikis et al. 2015).

The in vivo imaging of fluorescence‐tagged BSA and EGFP‐PMN on the flank of mice with skin wounds was performed using a commercial whole animal fluorescence imaging system (Xenogen IVIS 100 system, Perkin Elmer Waltham, MA), as previously described (Kim et al. 2008). Mice were put into the imaging chamber of the system after being anesthetized by ketamine‐xylazine. EGFP‐PMN within the wound area were visualized using a GFP filter (excitation at 445–490 nm and emission at 515–575 nm) at an exposure time of 1 sec. Simultaneous imaging of BSA‐AlexaFluor 680 within the wound area was achieved with BSA‐AlexaFluor 680 with a CY5.5 filter (excitation at 615–665 nm and emission at 695–770 nm, at an exposure time of 1 sec). BSA‐Alexa 594 within the wound area was imaged using DsRed filter (excitation at 500–550 nm and emission at 575–650 nm, at an exposure time of 1 sec). Analysis of the images was performed using Live Image Pro. 2.5 software (Caliper Life Sciences, Hopkinton, MA), and fluorescence intensities expressed as average radiance (photons per cm^2^ per steradian) were measured by drawing a circular region of interest over the entire wound area. Figure 1 illustrates the mouse imaging methods. Fluorescently labeled albumin extravasation into skin wounds was detected after intravenous tail vein administration of fluorescence‐conjugated albumin as a tracer. Apparent albumin permeability (*P*) was quantified based on the previously described linear relationship between the measured fluorescence intensity (*I*) and the concentration of fluorescently conjugated albumin within the wound area. *P*_s_ = 1/Δ*I*(0) (d*I*/d*t*) (*r*/2), where Δ*I*(0) is the step increase in fluorescence of tracer molecules due to vascular filling, d*I*/d*t* is the rate of albumin extravasation into the surrounding tissue, and *r* is the average radius of the postcapillary venules. As previously described (Kim et al. 2009), a value of *r* = 12.5 *μ*m was used as a median value for postcapillary venules with diameters 10–40 *μ*m. To a first approximation, the method accounts for changes in surface area for exchange provided the local microvascular plasma volume to microvessel exchange surface ratio remains constant. This is assumed to be valid even under conditions of vasodilation or vasoconstriction contemporaneous with shifts in tissue permeability as previously described (Curry et al. 2010).

### Measurement of albumin permeability coefficient and hydraulic conductivity in individually perfused rat mesenteric venules

Our well‐established methods in rat microvessels were used for these experiments, as there are very few microvessels in mouse mesentery suitable for microperfusion. The methods have been described in detail elsewhere (Curry et al. 2012). Briefly, the mesentery was continuously superfused with Ringer's solution (35–37°C). Experiments were performed on straight nonbranched segments of venular microvessels typically of 25–35 *μ*m diameter. The mesentery was observed via transillumination using a 20× (0.5 numerical aperture) long‐working distance lens (Zeiss Axiovert 100M microscope, Dublin, CA) and venular microvessels cannulated using a double‐barreled (*θ*) pipette to enable perfusion with either a control (non‐fluorescent) solution containing BSA (10 mg/mL) and red cells 1.5% hematocrit to maintain normal baseline permeability (Curry et al. 2012), or the same perfusate containing Alexa Fluor 595‐labeled BSA (0.5–1 mg/mL). Fluorescent intensity (*I*_f_) was measured using a high‐sensitivity camera (Hamamatsu EM CCD C9100, Hamamatsu, Japan) every 500 msec (30 msec exposure) and saved for offline analysis. Apparent *P*_s_ was calculated as above from the expression: *P*_s_ = [1/Δ*I*(0)] (d*I*/d*t*) (*r*/2). Hydraulic conductivity (*L*_p_) was measured using the modified Landis technique, which measures the volume flux of water crossing the wall of a microvessel perfused via a glass micropipette following downstream occlusion of the vessel (Michel et al. 1974; Kendall and Michel 1995; Michel and Curry 1999; Adamson et al. 2010; Curry et al. 2015). The initial transcapillary water flow per unit area of the capillary wall (*J*_v_/*S*)_0_ was measured at predetermined capillary pressures of 30–60 cmH_2_O. Microvessel *L*_p_ was calculated as the slope of the relation between (*J*_v_/*S*) and applied hydraulic pressure. *L*_p_ was estimated from each occlusion with the assumption that the net effective pressure determining fluid flow was equal to the applied hydraulic pressure minus 3.6 cmH_2_O, the approximate oncotic pressure contributed by the BSA in all perfusates. Experimental reagents were added to the superfusate and delivered continuously during both *P*_s_ and *L*_p_ measurement.

### ANP delivery to wound: estimate of effective concentration

In previous investigations using exogenous ANP in mouse, we and other investigators have infused ANP at a rate of 0.5 ng/g BW/min (500 ng/kgBW/min) i.v. to maintain a plasma concentration close to 1 ng/mL (Sabrane et al. 2005; Lin et al. 2011). Thus, the average whole‐body degradation of ANP in normal conditions appears to be close to 0.5 ng/g BW/min. Assuming skin and muscle are 40% body weight, this degradation rate is 1.25 ng/g/min in these tissues. In this experiment we added ANP directly to the wound at rates between 1 and 1.5 ng/min (mean 1.25 ng/min; applied as 6 ng in 10 *μ*l every 5 min). The average ANP concentration in the wound depends on the distribution volume for the added ANP, and the actual rate of degradation of ANP in the wound (which we expected to be higher than the normal average because of the inflammatory conditions). If the rate of degradation of ANP in the wound (6 mm diameter and ~300 *μ*m depth) is between 2 and 10 times normal, the average ANP concentration is predicted to fall in the range ~3–30 ng/mL (1–10 nmol/L). This range is identical to that previously used to record reduced leukocyte deformability and endothelial arrest in vitro (Morikis et al. 2015). Circulating ANP concentrations are markedly elevated under pathophysiological conditions (Joseph et al. 2013).

Atrial natriuretic peptide was added to the superfusate for all experiments in individually perfused rat mesentery microvessels. When low ANP concentrations (1–10 nmol/L) had no effect to modulate histamine responses, we increased ANP to the much higher concentrations (100–1000 nmol/L) to test the same range over which in vitro experiments with ANP demonstrated modulation of histamine responses (Furst et al. 2008; Chen et al. 2013).

### Analysis and statistics

The nonparametric Mann–Whitney test was used to compare pairwise unmatched groups having unknown variances. For repeated measures data we used the nonparametric Friedman test. For the comparison of a single group to a null hypothesis the *F*‐test was used. Where matched pairwise data were available, a nonparametric Wilcoxon test was applied. In all cases, significance was assumed for probability of less than 0.05. Averaged values are reported throughout as mean ± SEM.

## Results

### Thirty minutes pretreatment with ANP attenuates the acute thrombin‐induced increase in permeability in a skin wound produced 24 h previous

**Figure 2:**
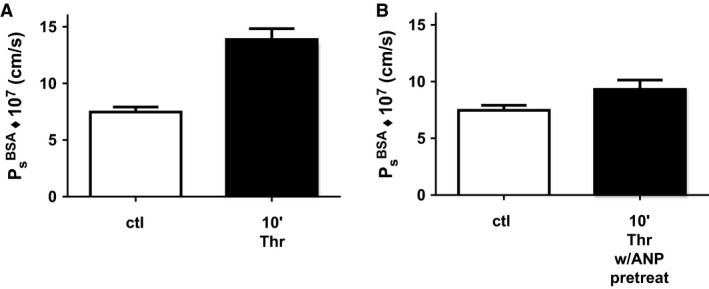
Atrial natriuretic peptide (ANP) attenuates the thrombin‐induced increase in apparent permeability coefficient to albumin. (A) Peak permeability at 10 min is significantly higher than control measured 24 h after the wound was formed (*n* = 9 Thr and *n* = 16 ctl; *P* < 0.05, Mann–Whitney). (B) After 30 min pretreatment with ANP (1.25 ng/min) directly into the wound the apparent albumin permeability coefficient is not significantly different from the control (*n* = 7 Thr/ANP and *n* = 16 ctl; *P* > 0.08; Mann–Whitney), but significantly less than thrombin alone (*n* = 9 Thr, *n* = 7ctl; *P* < 0.05; Mann–Whitney).

Baseline permeability to albumin in the vasculature surrounding a full‐thickness skin wound was first measured with AlexaFluor 595 dye labeled albumin, resulting in a mean control *P*_s_^BSA^ of 7.5 ± 0.4 × 10^−7^ cm/sec. Thrombin (5 U/mL) was then perfused directly into the wound and albumin labeled with AlexaFluor 680 was i.v. injected simultaneously. Figure 2A depicts the thrombin‐induced increase in apparent permeability coefficient to BSA (*P*_s_^BSA^) in a skin wound produced 24 h previous. The peak *P*_s_^BSA^ after 10 min exposure to thrombin was increased by 1.8 ± 0.1 times relative to control (13.9 ± 1.0 × 10^−7^ cm/sec, *n* = 9; *P* < 0.05, Mann–Whitney).

Pretreatment of the wound with ANP (1.25 ng/min) for 30 min followed by 10 min of perfusion with thrombin resulted in a mean *P*_s_^BSA−680^ of 9.3 ± 0.8 × 10^−7^ cm/sec (Fig. 2B). This value was not significantly different from control values measured prior to ANP treatment or ANP plus thrombin (Mann–Whitney) and was significantly less than the thrombin‐induced *P* ^BSA^ in the absence of ANP (Fig. 2A, Mann–Whitney). In additional experiments when ANP was washed out for 30 min after combined treatment with ANP and thrombin, *P* ^BSA^ was 15.1 ± 2.6 × 10^−7^ cm/sec indicating that the action of ANP to attenuate the thrombin response was reversible. Furthermore, a pretreatment period of at least 30 min with ANP was required in order to observe attenuation of the thrombin‐induced increase in permeability. This was confirmed in six additional experiments where ANP was added at the same time as thrombin, the action of ANP to attenuate the thrombin‐induced increase in *P* ^BSA^ was not statistically different from *P* ^BSA^ with thrombin alone.

### ANP acutely increases vascular permeability under the same conditions that it attenuates thrombin‐induced increase in permeability

**Figure 3:**
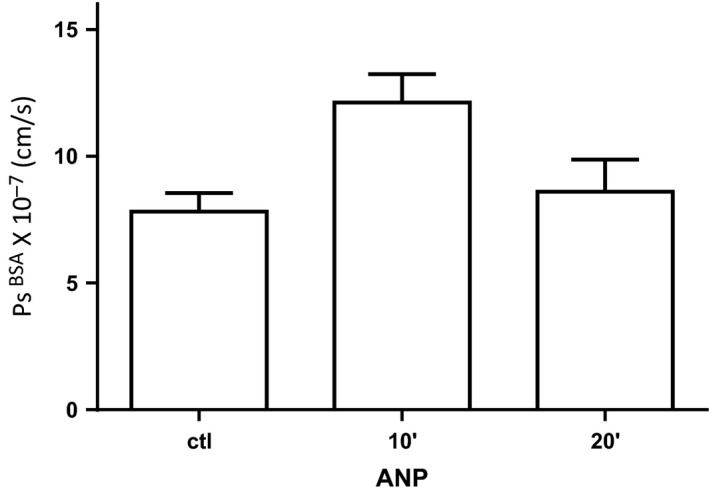
Measurement of apparent albumin permeability coefficient during the 30‐min period after atrial natriuretic peptide (ANP) is applied to the wound before testing the response to thrombin (Fig. 2B). ANP increases the albumin permeability after 10 min in the 24‐h skin wound (*n* = 12, *P* < 0.05; Friedman multiple comparison test) to the same extent as previously demonstrated in uninjured skin (Curry et al. 2010).

To test the hypothesis that ANP stabilized the endothelial barrier to changes in permeability, we measured *P* ^BSA^ in the wound when ANP was applied to a skin wound and no thrombin was added. The experiments in Figure 3 show that ANP (1.25 g/min) treatment alone induced an increase in apparent permeability to BSA‐Alexa680 (*P*_s_^BSA^) in mouse skin wounds made 24 h previous. Baseline permeability in the skin wound vasculature measured with Alexa 595 labeled albumin was 7.8 ± 0.7 × 10^−7^ cm/sec. ANP was then applied to the wound and Alexa 680 labeled albumin was injected simultaneously. The peak *P*_s_^BSA^ after 10 min exposure to ANP (12.1 ± 1.1 × 10^−7^ cm/sec) was significantly increased relative to controls (Friedman test, with Dunn's multiple comparison post‐test, *P* < 0.05 different from control, *n* = 12). Albumin permeability returned to control values over ~20 min. There was no significant difference between the initial control permeability and that measured at the end of the ANP application (20 min). Thus, ANP increases the permeability of microvessels in wounds under the same conditions that preconditioning by ANP attenuated thrombin‐induced permeability. Thus, there was no evidence that ANP stabilized the vascular wall to changes in permeability by known mechanisms such as an increase in intracellular cAMP.

The fractional increase in *P* ^BSA^ induced by wound treatment with ANP was similar to that elicited by ANP in the mouse skin without a wound (Curry et al. 2010; Lin et al. 2011). In fact in four additional animals pretreated for 30 min with the phosphodiesterase 4 (PDE4) inhibitor rolipram 8 mg/kg, ANP failed to increase permeability. This suggested the ANP increased permeability was not associated with a local increase in cAMP, but was more likely associated with decreased intracellular cAMP as previously reported by Ladetzki‐Baehs et al. (2007). The fact that we found no evidence that the endothelial barrier was stabilized by ANP, taken with our previous observations that ANP attenuated thrombin‐induced PMN accumulation, indicated that at least part of the action of ANP in attenuation of thrombin‐induced permeability increase depicted in Figure 2 was attributable to a reduction in leukocyte/endothelial interaction in the vasculature surrounding the wound.

### ANP reduces PMN numbers accessing the wound

**Figure 4:**
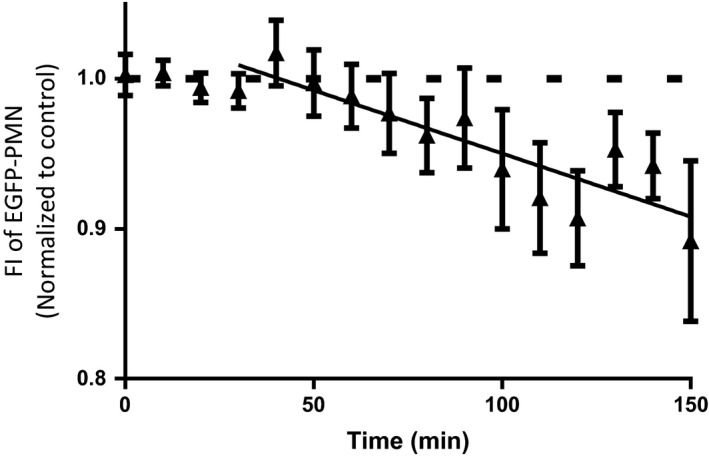
Atrial natriuretic peptide (ANP) induces the FI intensity from polymorphonuclear leukocytes (PMNs) in the skin wound to fall by 5%/h when applied to the wound. This is significantly greater than the result expected if ANP did not reduce PMN levels (*n* = 5; *P* < 0.0001; *F*‐test). We have previously demonstrated that after the 30 min of such exposure, ANP also attenuates thrombin‐induced hyperpermeability (as in Fig. 2B). The results in Figure 4 taken with these previous observations confirm that ANP reduces PMN accumulation in both the presence and absence of thrombin. Note that the FI reflects signal from all PMNs (intravascular and extravascular in the wound). To the extent that PMNs closely associated with the endothelial cell surface are only a fraction of the total PMNs, the fractional reduction in PMNs at the endothelial surface is expected to be larger than 10%.

In 3 of the 12 experiments in Figure 3 we simultaneously measured EGFP‐PMN fluorescence in the wound of Lys‐M‐EGFP mice (515–575 nm window) and albumin permeability (>680 nm window). Likewise, Figure 4 depicts the change in fluorescence intensity measured from PMN recruitment over a period of 120 min for these three experiments plus two additional experiments in which permeability was not measured (fluorescent albumin was not present). In response to wounding and in the absence of added ANP, the EGFP‐PMN signal was stable over the 30‐min control. Following ANP addition, the EGFP‐PMN signal fell steadily over 90 min. The reduction in FI intensity in the presence of ANP fell at a rate of` 5% per hour relative to the baseline value. This decrease was significantly different from the stable control level (0% per hour; *P* < 0.0001, *F*‐test). Thus, ANP acts to reduce the number of PMNs recruited to the wound (Fig. 4) under the same conditions as it acts to transiently increase vascular permeability (Fig. 3). These data conform to the hypothesis that the action of ANP to attenuate thrombin response (Fig. 2) is primarily by reducing PMN‐dependent mechanisms that increase permeability following their arrest on endothelium and associated platelet activation (Kim et al. 2009), rather than a primary action of stabilizing the endothelial barrier.

### Thirty minutes pretreatment with ANP does not attenuate histamine activated acute increases in vascular permeability in individual microvessels of rat perfused mesentery

**Figure 5:**
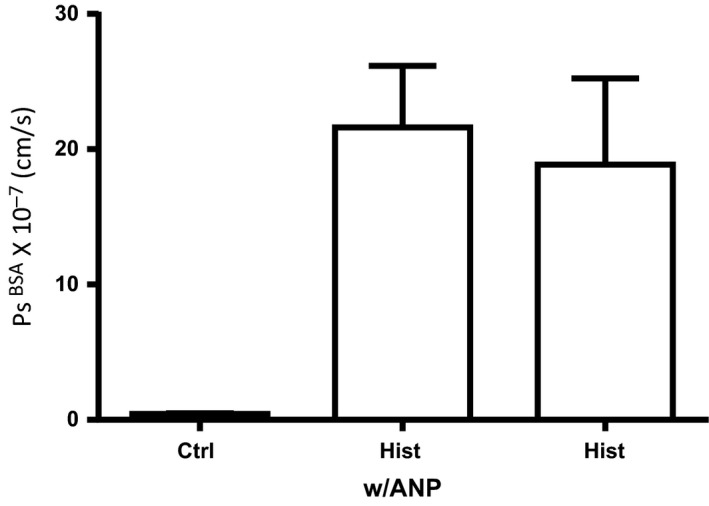
Atrial natriuretic peptide (ANP; 100 nmol/L) did not attenuate the increase in apparent albumin permeability (measured as peak response of Hist w/ANP relative to Hist alone) in response to histamine in individually perfused rat mesentery microvessels (*n* = 16 ctrl, *n* = 8 Hist w/ANP, *n* = 8 Hist, *P* = 0.64; Mann–Whitney). The perfusate in these vessels was a standard Ringer‐albumin solution containing red cell (1.5% to ensure a stable baseline permeability; Curry et al. 2012) but no polymorphonuclear leukocytes.

To test if ANP modulated increased permeability when leukocytes were absent, we measured *P*_s_ albumin in individually perfused microvessels. In 16 microvessels perfused with 1% BSA with added red cells, the baseline permeability to albumin (595 Alexa label) was 4.3 ± 0.6 × 10^−7^ cm/sec. Eight of the vessels were exposed to 100 μmol/L histamine directly after control measurements, and eight were exposed to ANP (100 nmol/L) for 30 min followed by histamine and continued ANP. Figure 5 shows that pretreatment with ANP did not attenuate the peak increase in albumin permeability in response to histamine (*P* = 0.64, Mann–Whitney).

Chen et al. (2013) reported that the effectiveness of ANP to attenuate histamine‐induced increases in permeability was potentiated by pretreatment with the PDE5 inhibitor sildenafil. Additional experiments with pretreatment of the microvessels with both ANP and sildenafil (1 μmol/L) did not change the response depicted in Figure 5. Moreover, the histamine response depicted in Figure 5 did not experience prolonged exposure to activated PMNs. The maximum time the individual microvessels were perfused was ~2 h. Histamine was used as an agonist instead of thrombin because we have previously reported that venular microvessels in rat mesentery only respond to thrombin activation after 24 h of exposure to inflammatory conditions. These experiments in individually perfused microvessels distinguish a role of ANP to modulate an increase in permeability under conditions where activated inflammatory cells are present for many hours (up to 24 h in our experiments). In contrast, the acute responses in perfused microvessels measured under conditions in which inflammatory cells are washed out of the vessel lumen and the endothelium is the primary target that lacks any discernable response to the presence of ANP.

### Thirty minutes pretreatment with ANP does not attenuate increased *L*_p_ with bradykinin and histamine

**Figure 6:**
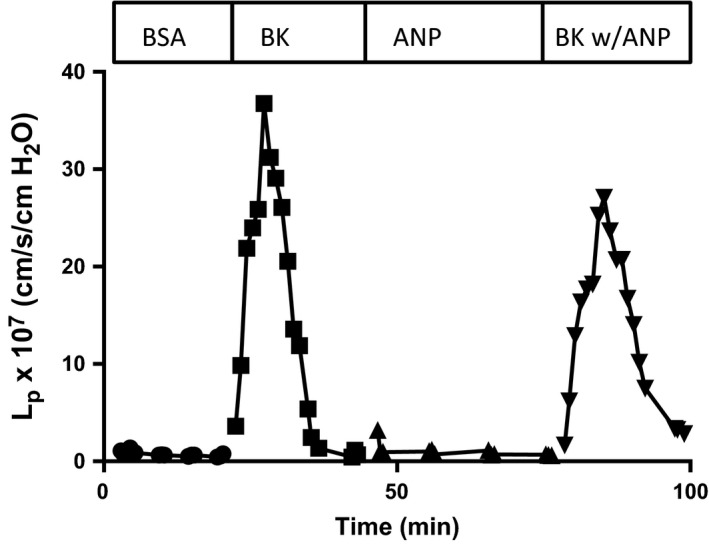
Representative experiment showing that atrial natriuretic peptide at concentrations up to 1000 nmol/L (as used in in vitro experiments) did not attenuate the increase in hydraulic conductivity after exposure of rat venular microvessels to bradykinin (10 nmol/L). The perfusate was as in Figure 5.

In a final set of experiments, we tested the action of ANP on acute increases in hydraulic conductivity (*L*_p_) induced by bradykinin and histamine. Here, we increased the concentration of ANP up to 1 μmol/L in order to match the conditions on cultured endothelial cells (Furst et al. 2008). In five vessels with 30 min of ANP pretreatment the mean baseline *L*_p_ was 0.65 ± 0.06 × 10^−7^ cm/sec/cmH_2_O and when exposed to bradykinin (10 nmol/L) the *L*_p_ increased to a mean of 18.0 ± 2.5 × 10^−7^ cm/sec/cmH_2_O. In the absence of ANP the mean baseline *L*_p_ with BSA was 0.76 ± 0.13 × 10^−7^ cm/sec/cmH_2_O and *L*_p_ increased to a peak value of 26.1 ± 5.9 × 10^−7^ cm/sec/cmH_2_O with bradykinin. Thus, as with measurements of albumin permeability, there was no significant effect of ANP to attenuate bradykinin (*n* = 5, *P* = 0.31; Wilcoxon). Figure 6 shows a single representative experiment, however, similar results were obtained using histamine in two separate groups of vessels. In microvessels with similar baseline values for *L*_p_ as in Figure 6, the response to histamine with ANP pretreatment (mean 14.3 ± 5.7 × 10^−7^ cm/sec/cmH_2_O; *n* = 7) is not different from the response in the absence of ANP (mean 12.2 ± 3.2 × 10^−7^ cm/sec/cmH_2_O; *n* = 9) (*P* > 0.9, Mann–Whitney test). Taken together, the data indicate that ANP did not attenuate the transient increase in *L*_p_ after exposure of the venular microvessels of rat mesentery to bradykinin.

## Discussion

We have demonstrated that after only 30 min pre‐exposure to ANP, the thrombin‐induced increase in vascular permeability is attenuated in a mouse skin wound. This observation is consistent with previous reports that ANP exerts an anti‐inflammatory action in vasculature induced to a high permeability state. The key new observations here are that during the 30 min pre‐exposure, ANP acts in the 24 h wound via at least two different mechanisms. On one hand, the well‐established action of ANP, acting via cGMP‐dependent mechanisms (Joseph et al. 2013) to increase vascular permeability to albumin is not significantly modified in the wound compared with the normal baseline state as demonstrated in Figure 3. On the other hand, ANP causes a steady decrease in the number of PMNs that accumulate in the wound in response to the inflammation induced by the wound injury. When combined with our previous observations that ANP reduces thrombin‐induced accumulation of PMNs in the 24 h skin wound, these observations support the conclusion that the primary action of ANP under the conditions of our experiment to attenuate the thrombin‐induced increase in permeability is not to stabilize the endothelial barrier, but to modify PMN‐dependent mechanisms stimulated by thrombin that increase vascular permeability. This result is consistent with our previous observations that ANP has a striking action to decrease PMN deformability and thereby attenuate the rolling, arrest, and transendothelial migration on cultured cell monolayers (Morikis et al. 2015). Our investigations unify the results of in vivo and in vitro investigations that point to the potential for ANP to modulate inflammatory responses by limiting PMN–endothelial interactions.

Our recently published in vitro investigations demonstrated that ANP reduced PMN deformability and their capacity to spread onto the endothelial surface. ANP treatment was observed to increase PMN cytoplasmic viscosity within minutes, whereas the subsurface membrane cortical tension remained constant (Morikis et al. 2015). Furthermore, single‐cell image analysis of PMNs rolling to arrest under fluid shear stress demonstrated a significantly smaller adhesive contact area and diminished formation of durable integrin bond clusters between leukocytes and endothelium. Cellular mechanisms that may modulate the reduced leukocyte deformability include ANP signaling via GC‐A receptors on leukocytes that activate Rac GTPase and its effector PAK1, which in turn downregulates GEF‐H1 activation via phosphorylation at Ser^855^. One downstream consequence is reduced activation of Rho‐GTPases, which are key regulators of actin dynamics in vascular cells (Damiano et al. 1996). Additionally, ANP has been shown to stabilize microtubules, leading to its vasoprotective effects (Tian et al. 2014). One or more of these mechanisms could account for our observation that ANP reduced PMN accumulation in the skin wound.

An important new result is that ANP does not have a significant effect to attenuate the acute responses to histamine and bradykinin when the perfusion conditions are controlled such that resident leukocytes that may roll along the vessel wall at the beginning of the experiment are washed away and the number of attached leukocytes is minimal (Curry et al. 2012). We tested ANP at concentrations up to 1000 nmol/L because experiments in cultured endothelial monolayers had reported inhibition of histamine‐induced increases in permeability under these conditions (Furst et al. 2008). Furthermore, continuous perfusion of the microvessels in the absence of circulating leukocytes is in contrast to the state of microvessels in previous reports using mouse cremaster muscle (Furst et al. 2008; Chen et al. 2013), which were continuously exposed to circulating blood. Under these conditions histamine is known to stimulate leukocyte–endothelial interactions (Yamaki et al. 1998). These differences highlight the need to evaluate mechanisms for the modulation of vascular permeability modulation at multiple levels of vascular organization. For example, they highlight important new questions about the interpretation of results under disparate experimental conditions.

Whereas our experiments revealed little action of ANP on endothelium stimulated with histamine in the virtual absence of leukocytes, previous investigators attributed a nearly 50% reduction in peak permeability response to the anti‐inflammatory action of ANP on histamine‐stimulated endothelial cells. Thus, Furst et al. (2008) appear to have ignored the contribution of ANP on leukocyte–endothelial interaction. On the other hand, Chen et al. (2013) demonstrated that the action of ANP to attenuate the histamine response in cremaster muscle microvessels was absent in mice with endothelium‐specific deletion of the cGMP generating GC‐A receptor for ANP or the endothelial cGMP‐dependent protein Kinase 1 (cGKI). Their data are clearly consistent with a significant action of ANP in these two KO mouse models. It remains to be determined if differences in the relative contribution of endothelial‐ and leukocyte‐dependent mechanisms can be explained by differences in experimental conditions (cremaster muscle vs. mesenteric venular vessel, FITC Dextran vs. albumin as a permeability probe, the presence of other blood components vs. Ringer with albumin and red cells). In addition, it is important to note that the mice lacking endothelial GC‐A and cGKI were generated using a Tie2/Cre‐Lox approach in mouse hematopoietic cells, an approach usually considered to enable endothelial‐specific mechanisms assuming Tie2 is an endothelium‐specific marker. However, the specificity of Tie2 promoters to direct gene regulation only in endothelial cells has recently been questioned because of the widespread expression of Tie2 in other inflammatory cells of hematopoietic origin including PMNs (Tang et al. 2010; Joseph et al. 2013). Thus, recent investigations measuring both leukocyte accumulation and endothelial cell response in mice in Tie2‐Cre mice point the way to further investigations, especially if the responses of PMN to ANP under the conditions of the experiments are suspect (Chen et al. 2016).

It is also noted that the same rat venular microvessels that respond to histamine and bradykinin do not respond to thrombin in individually perfused microvessels (Curry et al. 2003). Thrombin causes increased permeability only after sustained exposure to inflammatory conditions. The same result is found in mouse skin (Kim et al. 2008). The present results indicate that this response is likely to involve PMN‐derived products stimulated by thrombin.

Our investigations do not rule out the possible action of ANP on endothelium under different experimental conditions. The observation of Beavo's group that ANP‐dependent increases in cGMP may either increase or decrease cAMP via cGMP‐dependent phosphodiesterase indicates that, depending on the relative expression levels of these PDEs, ANP may exert either an increased permeability by lowering intracellular cAMP or lowered permeability when intracellular cAMP levels are increased (Surapisitchat et al. 2007). Our observation that the PDE4 inhibitor rolipram attenuated the ANP‐induced permeability can be explained if the action of rolipram to independently increase cAMP overrides actions of ANP to reduce cAMP levels under the conditions of our experiments (Lin et al. 2012). Consistent with such an action of ANP, we have demonstrated that mice lacking cAMP‐dependent Epac1 (Epac1−/− mice) do not respond to ANP through an increase in permeability but have elevated baseline permeability (Kopperud et al. 2016). One example of the way that changes in the expression level of PDEs in endothelium modulates responses to ANP is the observation that mice with EC‐specific deletion of their ANP receptor (GC‐A) have mildly but significantly smaller infarct size compared to control littermates (Chen et al. 2016) after permanent ligation of the proximal part of their left coronary artery. These authors show that ANP may fail to protect the heart under these conditions because, in response to TNF‐*α* released in the heart after the ligation, the expression levels of cAMP degrading PDE2A are increased. The result is a lower level of cAMP concentration close to the endothelial cell membrane in the control animal (with endothelial ANP receptors) than in the ANP receptor KO mice. Although endothelial actions of ANP signaling appear to have been more important than its action on PMN in these mouse models (PMN accumulation after 2 days is similar in both control and KO mice), the levels of expression of the ANP receptor on PMN in both KO and control littermates and the way ANP modulates the interaction of these PMN with endothelium has not been systematically investigated.

We note that to make measurements of both permeability and PMN accumulation under similar conditions required the use of a combination of whole animal and microperfusion experiments in rats and mice. No rat model was available where the PMNs‐expressing EGFP could be used to quantify their accumulation under the same conditions as permeability was measured and the mouse tissues (mesentery and muscle) have very few microvessels suitable for microperfusion. Furthermore, we did not investigate acute responses to thrombin in perfused microvessels, as thrombin does not acutely increase permeability in vessel that has not been exposed to inflammatory conditions. Thus, in addition to our specific goal to investigate ANP‐dependent regulation of vascular permeability with and without the presence of leukocytes, these investigations (in parallel with our recent in vitro investigations) demonstrate some of the current strengths and weaknesses of current strategies to understand the modulation of vascular permeability at the cellular, single microvessel, and whole microvascular bed approaches.

On the basis of our results we predict that the effectiveness of the vasoprotective actions of ANP likely depends on the nature of the primary microvascular dysfunction and activation state of the endothelium. ANP is not expected to be protective when the primary insult is an acute inflammatory agent which acts via mechanisms in endothelium similar to histamine (calcium influx, reduced endothelial cell adhesion, gap formation in microvascular endothelial cells; Chen et al. 2013) and where the net effect of ANP action in endothelial cells is reduced submembrane cAMP levels. On the other hand, ANP is expected to be more protective under conditions in which leukocyte/endothelial interactions are promoted and ANP modulation of leukocyte deformability effectively reduces their accumulation. In experimental models and in clinical situations both inflammatory stimuli are likely to be present and the outcome dependent on which ANP‐dependent pathways modulate endothelium and leukocytes.

## Conclusion

The hypothesis that the anti‐inflammatory actions of ANP result solely from ANP ligation of endothelial receptors that act to stabilize barrier function is not supported by our results in either individually perfused microvessels in the absence of circulating leukocytes, or the more chronic skin wound model. Our results conform to an alternate hypothesis that ANP modulates the interaction of leukocytes with the microvascular wall in the inflammatory state of a 24 h wound. The action of ANP to attenuate leukocyte–endothelial interactions and attenuate a subsequent thrombin‐induced permeability increase in the presence of accumulated PMNs is corroborated by our previous report that ANP decreases leukocyte deformability, thereby reducing the adhesive contact area and strength of attachment to endothelium. These observations provide the basis for additional investigations of the use of ANP as an anti‐inflammatory agent to modulate leukocyte–endothelial cell interactions.

## Conflict of Interest

None declared.
